# Personality traits in free-ranging dogs: Do experimental tests mirror natural behavior?

**DOI:** 10.1016/j.isci.2025.113856

**Published:** 2025-10-25

**Authors:** Urša Blenkuš, Friederike Range, Debora Prince, Corisande Abiven, Giulia Cimarelli, Sarah Marshall-Pescini

**Affiliations:** 1 Domestication Lab, Konrad-Lorenz-Institute of Ethology, University of Veterinary Medicine Vienna, Savoyenstrasse 1a, Vienna 1160, Austria; 2 Department of Chemistry, Life Sciences and Environmental Sustainability, University of Parma, Viale Delle Scienze 17/A,Parma 43124, Italy; 3 Behavioural Ecology Group, Wageningen University & Research, De Elst 1, Wageningen 6708, the Netherlands

**Keywords:** Canine behavior, Natural sciences, Biological sciences

## Abstract

While animal personality research is expanding, methods used to assess personality traits of free-ranging animals are often unreliable or report mixed results in the validation process. One potential reason is inadequate implementation of methods used for captive animals to wild populations. In this study we assessed cross-context validity of human- and conspecific-directed sociability, exploration, and aggression in a free-ranging dog population, with measures obtained from experimental testing and naturalistic observations. We found strong agreement between the two methods for human-directed sociability and exploration, supporting their validity. Although conspecific-sociability showed limited associations, initial reactions to other dogs during the test was linked to conspecific proximity during observations. Aggression occurred rarely, hence was not evaluated. Our results show that robust personality assessments are possible in free-ranging dogs, even in challenging field environments, paving the way for future studies, to advance our understanding of the species’ behavioral ecology.

## Highlights

• Personality assessment in wild animals is challenging and methods lack validation
• We assess personality in free-ranging dogs through experiments and observations
• We measure exploration, human and conspecific directed sociability
• We found a good match between observational and experimental methods

## Graphical abstract

**Figure:**
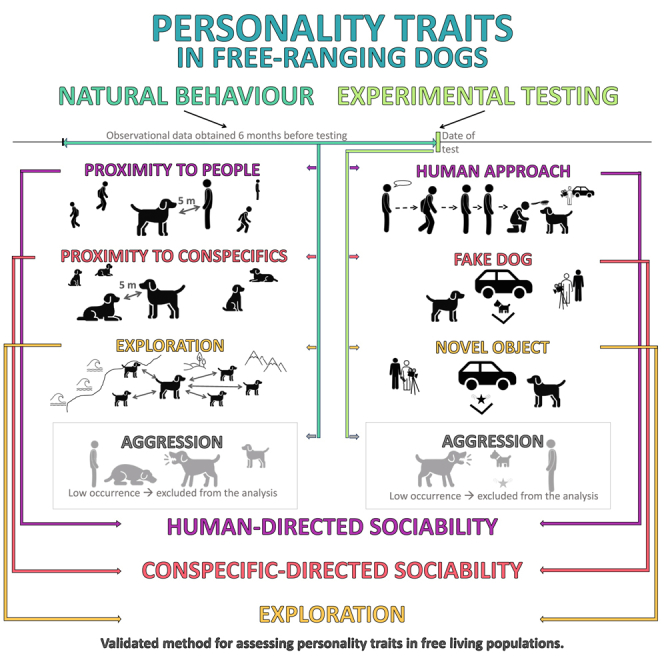

## Introduction

Personality—also referred to as temperament, or coping style—is defined as a set of behavioral traits that are both temporally stable and repeatable across contexts.^1^^,^^2^^,^^3^^,^^4^ While genetic variation does have a role in the development of behavioral traits,^5^ the ecological and social niche can also increase the expression of certain environmentally preferred behaviors over others,^6^ shaping individuals’ behavioral phenotypes and potentially leading to differentiation between populations.^7^ For example, for a number of species, populations living in urban areas appear to be more explorative than those living in rural areas (mice [Apodemus agrarius],^8^ lizards [Anolis sagrei],^9^ great tits [Parum major]^10^). Boldness was observed to have a positive impact on survival in European mink (*Mustela lutreola*),^11^ but negative impact on survival was observed in spotted hyaenas (*Crocuta crocuta*). Due to the interaction between environment and personality and the respective impact on fitness, studying personality in animals’ natural environment is paramount to understand its evolutionary importance.

To date, the majority of animal personality research has been carried out in laboratory or zoo settings, with far fewer studies being performed in the wild.^12^^,^^13^ Due to this focus, animal personality research usually relies on methods developed in laboratory settings, where control over environmental variables is higher.^14^ In such environments, mainly two approaches have been used to study animal personality: (1) “experimental testing”—during which a subject is presented with a set of stimuli and its behavioral responses are recorded, and (2) “trait rating”—where an observer familiar with the subject rates the individual based on a set of predefined, questionnaire-based items. This latter approach is generally done using knowledge of how the animal behaves in its everyday life, although this method can also be applied during experimental tests.^13^^,^^15^^,^^16^^,^^17^^,^^18^^,^^19^

Conducting personality assessments on wild animals poses additional challenges and has been done using “naturalistic observations”—where the spontaneous behavior of the animals is recorded during their daily activities. These observations are time consuming, hard to account for cross-situation consistency and require animal recognition. Yet, they provide us with a larger dataset with continuous measurements for each individual.^16^ Experimental testing is used as another approach for personality assessment of wild animals, although, for this purpose animals usually have to be captured and relocated,^13^ which may inadvertently bias results, with bolder animals being more likely to be captured than shyer ones, skewing the representation of phenotypes within the given populations.^20^ Furthermore, the impact of relocating the animals from their natural habitats remains insufficiently understood^13^^,^^21^ with stress-induced alterations in behavior possibly undermining data validity.^22^ An alternative approach is to use lab-designed experimental procedure and adapting them to the field environment, avoiding the need for capture and relocation of wild animals, (free-ranging dogs [*Canis familiaris*],^23^ fox squirrels [*Sciurus niger*]).^24^ However, even without capturing and relocating the animals, it is unclear to what extent behaviors measured under testing conditions where humans are present and the animals are restricted, adequately reflects their natural-occurring behavior. In light of this, wherever possible cross-context comparison of behavioral traits should be implemented, to assess the measures’ reliability and validity.^13^^,^^16^^,^^21^

Only few studies have incorporated this approach, and results are mixed. Carter et al.^17^ found that boldness in wild chacma baboons (*Papio ursinus*) was reliably measured both with observational methods and trait rating, while mismatch between experimental and observational methods was reported by Tkaczynski and Ross^18^ for barbary macaques (*Macaca sylvanus*). Studies with wild red squirrels (*Tamiasciurus hudsonicus*) have reported a mismatch between experimental and observational methods,^25^ while correlation was observed between exploration in experimental setting and foraging in observational setting.^26^ Krebs et al.^27^ observed that wild house mice (*Mus musculus domesticus*) exploration/activity measured with standardized behavioral tests was negatively correlated with exploration/activity of the same individuals in a semi-natural environment. To reconcile all approaches, it has been suggested that each method (i.e., experimental or observational) can be used for different purposes. Experimental methods have been suggested to be a better measure for assessment of non-social parameters, that occur rarely and unpredictably in naturalistic observations^28^ and can be controlled to a greater degree,^25^ while observations have been suggested to be better at assessing social parameters^28^ and have higher ecological validity.^18^ Yet, additional research is needed to better understand to what extent the different methodologies are able to capture the same behavioral patterns, while at the same time expanding the range of species of interest.

Domestic dogs (*Canis familiaris*) are perfect candidates to investigate whether natural observations reflect measurements in experimental settings and vice versa. On the one hand, as pet dogs, they give researchers a unique opportunity to test them in standardized and strictly controlled conditions using experimental testing,^29^ as well as to observe their everyday spontaneously occurring behaviors using trait rating.^30^ On the other hand, free-ranging dogs, exist in and around human settlements, and while they are free to roam and reproduce, they are often dependent on food provided by humans.^31^ Differently from previously thought, free-ranging dogs can be considered mongrels from the genetic point of view and are not an admixture of different breeds.^32^ Thus, they provide us with a unique opportunity to test dogs in an environment, where natural and sexual selection are still ongoing processes.^31^ and animals have not been artificially selected for their behavioral and morphological traits as pet dogs usually are. This can allow us to better understand what socio-ecological pressures shape dogs’ natural behavior. Thanks to the long domestication process and their natural environment being in proximity to humans, free-ranging dogs give us the possibility to approach them without the need to capture or restrain them, as compared to other wild animals, thus reducing the potential biases inherent in testing wild animals, where human presence and a natural shyness toward them might hinder the possibility to reliably detect personality differences by using field experiments. Indeed, short experimental procedures, involving the voluntary participation of free-ranging dogs aimed at measuring free-ranging dogs’ behavior and cognition have already been successfully conducted in field conditions, during experimental testing^33^^,^^34^ and trait rating.^35^

Building on this approach, in a previous study we developed a behavioral test battery to measure human- and conspecific-directed sociability, neophobia, dog-human communication, and tractability in free-ranging dogs.^36^ Results showed good reliability and temporal stability for the traits of interest.^36^ To further validate this new method, in the present study, we evaluated the cross-context validity of our newly designed behavioral test battery (BTB), with measures of the same traits extracted from the observation of the animals’ everyday spontaneous behaviors, based on proximity scans (PS). Tests of interest from BTB were human approach (HA), fake dog (FD) and novel object (NO) test, while from PS we assessed personality traits based on dogs’ movement in the environment and their spontaneous behaviors. Due to testing conditions in disturbance-prone environment, we first ensured that the data collected with each method showed good inter-rater reliability and temporal stability (see STAR Methods and Documents S3-S5). Personality traits from BTB were obtained by running a principal-component analysis (PCA) analysis (Document S6) from behaviors observed during testing, while for PS personality traits were observed through continuous encounters with the dog over a 6-month period prior to BTB test (see STAR Methods). If our test reliably measures personality characteristics of our study population, we predict that the same individuals will show a similar pattern of responses for each behavioral trait, regardless of the assessment method used. Association between the two was assessed by running a model for each trait of interest (see STAR Methods and Document S7).

While terminology in the field is not unified, in this paper we use the categorization of personality traits as suggested by Réale et al.^2^: (1) exploration-avoidance, as an individual’s reaction toward novelty or exploration of a new environment, (2) aggressiveness, as an individual’s agonistic reaction toward conspecific, and (3) sociability, as a measure of how inclined an individual is to seek or avoid the presence of conspecifics. While sociability is usually measured as an individuals’ propensity to seek out or avoid conspecifics, we here include the additional aspect of (4) human-directed sociability, as humans represent an integral part of free-ranging dogs’ social environment.^33^^,^^37^ Through a long domestication process, dogs have developed a self-driven motivation to interact with humans.^38^ Human-directed sociability therefore emerges as a personality trait in studies of pet dogs using both behavioral testing and trait rating.^39^^,^^40^

Although differentiation between shyness-boldness and exploration-avoidance is not well defined,^2^^,^^13^^,^^21^^,^^41^ in the current study, because the free-ranging dogs we observe are free to move in their environment and their participation in the behavioral test battery is voluntary, we consider their involvement in our test as an expression of their curiosity, and view it as part of the exploration-avoidance axis.^21^ Thus, based on the behavioral test and the observation of the dogs’ naturally occurring behaviors, we assessed the cross-context validity of the following traits: human- and conspecific-directed sociability, exploration, and aggression.

By developing and validating reliable methods to measure these personality traits in an animal’s natural environment, we aim to provide tools that will allow researchers not only to investigate how personality is affected by different socio-ecological constraints, but also to investigate how personality may affect group social dynamics in social species.

## Results

### Human-directed sociability

**Figure 1:**
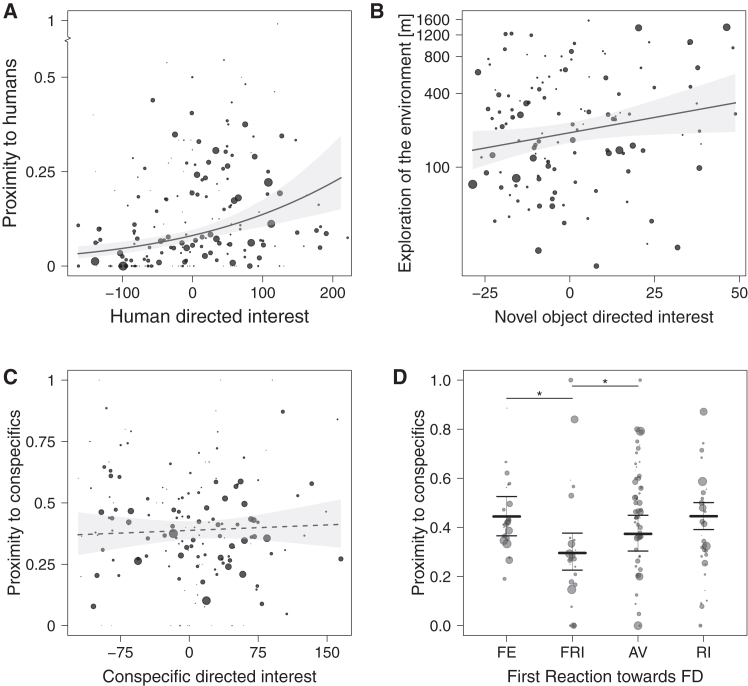
Graphical presentation of the link between human-directed sociability, exploration, and conspecific-directed sociability in the BTB and PS (A) Graphical presentation of human-directed sociability (*p* < 0.001—binomial model). (B) Exploration (*p* = 0.032—linear model). (C) Conspecific-directed sociability (non-significant, *p* = 0.335—binomial model). (D) Conspecific-directed sociability as measured with the dogs’ First reaction to the fake dog (*p* = 0.014—binomial model). For human-directed sociability, exploration, conspecific-directed sociability line presents predicted values of the model, with gray area indicating 95% confidence intervals. Values on the *x* axis present PC1 scores from BTB. For First reaction toward conspecifics predicted values are presented with the middle line and 95% confidence levels with upper and lower line. Number of times the dog has been seen during the scan is presented with the size of the dots. Note: Sociability toward humans has a break on the *y* axis. Exploration has exploration of the environment presented as log transformed value. (∗ - *p* < 0.05—binomial model, FE—fearful approach, FRI—friendly approach, AV—avoidance, RI—rigid approach).

Higher levels of human-directed interest during BTB (PC1 component of the HA; 33.23% explained variance, comprising of: sniffing person, body contact, tail wagging toward E1, first approach, and close proximity to E1; Table S11) were positively associated with proximity to humans during PS (cross-context reliability model, effect of human-directed interest 0.48 ± 0.10, χ2 = 21.3, df = 1, *p* = 0.001, lower CI 0.28-upper CI 0.68, Figure 1A, for details see Document S7).

### Conspecific-directed sociability

The full model, comparing conspecific-directed interest (PC1 component of the fake dog (FD); 34.83% explained variance, comprising of: close proximity toward FD, sniffing FD, gazing toward FD, latency to approach FD, and tail wagging toward FD; Table S12) and first reaction toward the fake dog during BTB with proximity to conspecifics during PS, was statistically significant, when compared to the null model (likelihood ratio test χ2 = 10.96, df = 4, *p* = 0.036, for details see Document S7). More specifically, conspecific-directed interest during BTB did not show a significant association to proximity to conspecifics during PS (Table 1; Figure 1C), while the first reaction toward the fake dog during BTB was observed to significantly predict proximity to conspecifics during PS (Table 1; Figure 1D)—dogs that displayed a friendly approach to the fake dog were less often seen in close proximity to conspecifics during PS as compared to dogs that approached the FD in a fearful manner (*p* = 0.033, Figure 1D) or avoided interactions with FD (*p* = 0.013, Figure 1D).

**Table 1:** Results of cross-context reliability between BTB and PS, for conspecific-directed sociability (estimates and standard errors, together with confidence limits, and significance tests)

	Estimate	St err	Lower CI	Upper CI	χ^b^	df	p^a^
Intercept	3.04	0.06	2.93	3.16	–	–	^(2)^
Conspecific-directed interest^c^^,^^d^	0.02	0.03	−0.04	0.08	0.93	1	0.335
FD First Reaction FRI^e^	−0.27	0.09	−0.45	−0.09	10.64	3	0.014
FD First Reaction RI^e^	−0.32	0.08	−0.48	−0.15			
FD First Reaction AV^e^	−0.33	0.08	−0.48	−0.18			

FE, fearful; FRI, friendly; RI, rigid; AV, avoidance

a the indicated significance test referees to the overall effect of the predictors.

b not indicated because of being of very limited interpretation.

c z transformed to a mean of 0 and standard deviation of 1; mean and sd of the original variable were 0.00 and 63.41, respectively.

d Conspecific-directed interest - BTB residuals were calculated as the residuals from a model with PC1 as a response variable, body condition class, temperature during BTB, activity level during FD, and sex as fixed effects (see Document S7).

e referenced based on FE.

### Exploration

Parameters of exploration between BTB (PC1 component of the NO; 32.41% explained variance, comprising of: tail wagging toward NO, vocalization (barking, growling, and whining), and gazing toward NO; Table S13) and PS were observed to be significant when comparing the full to the null model (likelihood ratio test F = 4.710, df = 1, *p* = 0.032). NO directed interest during BTB and Exploration of the environment as observed in the PS showed a significant association, with dogs that expressed higher interest in the NO being more likely to explore their environment (cross-context reliability model, effect of NO directed interest 0.21 ± 0.03, t = 2.17, df = 1, *p* = 0.032, lower CI 0.02-upper CI 0.41, and effect of NO activity level −0.11 ± 0.09, t = −1.08, df = 1, *p* = 0.282, lower CI −0.31-upper CI 0.09, Figure 1B, for details see Document S7).

### Aggression

The number of individuals showing aggressive behaviors (biting, growling, bare teeth, and lunge) during the BTB (20 out of 201 dogs) and the PS (33 out of 201 dogs) was too low to perform any further analysis. 154 dogs showed no signs of aggression during both tests, 27 dogs showed aggressive behavior during PS but not during BTB, 14 dogs showed aggressive behavior during BTB but not during PS, and 6 dogs showed aggressive behaviors during both tests.

## Discussion

The goal of our study was to assess whether personality measures of free-ranging dogs taken in an experimental context reflect the spontaneous behaviors exhibited by the animals in their everyday life, assessed with observational measures. Four distinct traits were identified from both PS and BTB: human-directed sociability, conspecific-directed sociability, exploration, and aggression. Human-directed sociability and exploration showed reliable cross-context validity between the two methods, thus confirming that experimental testing reflects the animal’s spontaneous behaviors. While for conspecific-directed sociability, although we observed some cross-context validity, this association needs to be better explored (see below). We were unable to test for aggression due to insufficient behavioral observations of aggressive behaviors in both contexts.

For human-directed sociability, we found a strong association between the human-directed sociability measures taken in the behavioral test battery and the spontaneous observations of the dogs’ vicinity to humans taken from the observational methods. Additionally, human-directed sociability had the highest temporal stability based on observational measures, confirming previous finding showing a high temporal stability in the experimental context.^36^ Together these results suggest the importance of this personality axis for free-ranging dogs. Indeed, previous studies have shown the importance of humans for free-ranging dogs, for example, in the survival of free-ranging puppies,^37^ as well as high number of interactions that adult dogs have with humans on a daily basis.^33^ Domestication has potentially enhanced the importance of human-directed sociability as a personality trait in dogs, given the latter’s high dependence on the former even in free-ranging situations.^42^ Furthermore, studies in captivity have shown that compared to wolves (*Canis lupus*), dogs show hypersocial behavior toward humans,^43^ are more likely to search for human proximity,^44^ and gaze toward humans more,^45^ which potentially indicate the effect of the domestication process on dogs’ human-directed sociability.

In contrast to results relating to human-directed sociability, results relating to conspecific-directed sociability were not in complete agreement with our predictions. When considering the dogs’ conspecific-directed interest during the whole fake dog test and the proximity to conspecifics during observations, we found no significant association. Although the fake dog test is a validated experimental approach to measure conspecific interactions,^46^ and our dogs’ behavioral responses to the fake dog were aligned with expected behaviors toward conspecifics observed also in other studies (e.g., dominance behaviors, piloerection, and genital sniffing^47^), it is unclear to what extent dogs perceive the fake dogs as a conspecific.^48^ It is possible that the fake dog is perceived as a conspecific in the initial moments of the encounter, but not for the full 2-min duration of the test. Thus, in this study, we addressed this issue by separately assessing subject’s first reaction toward the fake dog as well as their behavior during the whole test. Interestingly, the dogs’ first reaction to the fake dog was observed to significantly predict proximity to conspecifics during observations. However, contrary to our expectations, dogs that exhibited fearful or rigid responses toward the fake dog during the behavioral test were more often observed in proximity to conspecifics during observations. While the fake dog test is intended to measure dogs’ response toward an unfamiliar dog, the observational measures recorded the dogs’ proximity both to familiar and unfamiliar dogs. We were unfortunately unable to distinguish from our records between the two, which could be a possible reason for this apparent discrepancy. Dogs that are part of a larger pack are those that are more often observed in close proximity to (familiar) conspecifics. These same dogs may show an increased likelihood of insecure behaviors when individually presented with an unfamiliar dog during the fake dog test, because they may be less used to encountering unfamiliar dogs without the support of other pack members. Taken together, these results suggest that the initial response to the fake dog test may indeed be associated with aspects of conspecific sociability, however, future work needs to distinguish more precisely between the levels of familiarity of dogs in order to complete the assessment of its validity.

Exploration is a commonly measured trait in wild animals,^13^ with assessment commonly being performed in an experimental setting, for example as exploration of novel environments (fairy-wrens [*Malurus cyaneus*]^49^), open field test (turtles [*Emydoidea blandingii*],^50^ mice [*Apodemus agrarius*],^8^ starlings [*Sturnus vulgaris*]^51^), or novel objects (orangutans [*Pongo* spp.]^52^). To ensure the ecological relevance of these methods, previous studies compared exploration measured in experimental settings, with animals’ exploration of their natural environment. While we observed that exploration of the NO correctly predicted exploration of the environment in our study, other studies found less clear results. Krebs et al.^27^ observed negative correlation when comparing exploration of wild house mouse (*Mus musculus domesticus*) during NO, open field and elevated plus maze with exploration and colonization of a semi-natural environment. This could be due to the unnatural organization of the environment, with all essentials (food, water, shelter) being present in a small area, reducing animals’ need to travel. Sanders et al.^26^ and Martinig et al.^25^ observed behavior of North American red squirrels (*Tamiasciurus hudsonicus*) during an open field test and compared it with traveling/movement and feeding/foraging, with almost no correlation observed between the behavioral axes. While in our experiment the animals were free to explore the NO but also leave the area if they so wished, in these studies the animals were restrained within an arena during experimental testing, which could have had an impact on the animals’ behavioral response. Tkaczynski et al.^18^ measured exploration in barbary macaques (*Macaca sylvanus*) during exposure to a NO in a group setting and compared it with behavioral traits obtained from focal observations. They did not, however, observe any clear behavioral traits that could be directly compared between the two methods, which could be the reason, why they were not able to observe clear correlations. Based on current results showing that environmental exploration and exploration of a NO when animals are unrestrained are related, we suggest these measures could provide an interesting avenue of research also for wild species.

Due to its low occurrence, both during BTB and PS, aggression (toward conspecifics and humans) had to be excluded from our analysis (BTB: 20 out of 201 dogs; PS: 33 out of 201 dogs). While aggression has specific benefits for the individual, such as securing mates, food, and space, it comes with the risk of injury or even death, physiological and psychological costs, and a reduction in the strength of social relationships in group living species.^53^ Therefore, depending on the context and species, it can be beneficial for individuals to generally avoid being aggressive. Free-ranging dogs live in proximity to humans, who do not tolerate dog aggression neither toward them nor toward other conspecifics (personal observations), with aggressive individuals receiving less food from people or even being removed from the population (in case of high levels of aggression). These human behaviors are an additional human-imposed selection for low levels of aggression in the population. The highest levels of dog-directed aggression were observed in specific context, such as mating or access to food that was not often represented in our data. For future studies, continuous observations of the individuals might be more appropriate for this purpose, as it increases the possibility to observe behaviors which are less frequently exhibited.^54^

To conclude, we demonstrated that both field experiments and observational methods can be used to assess human-directed sociability and exploration in free-ranging dogs in a reliable way. Conspecific-directed sociability shows promising results, but it needs to be interpreted with caution, hence future investigations should consider potential differences in social behavior toward more or less familiar individuals. Based on the current cross-context validation showing how experimental procedures may accurately reflect an animal’s spontaneous behaviors, we suggest that either method can be used depending on the feasibility in a given population, since both methods provide reliable and valid data in characterizing key personality traits. By validating this method, we paved the path for further studies aiming at asking additional questions regarding animal personality, such as the expression of personality traits in different environments, its fitness consequences, the expression of traits within a group composition, and other aspects that would increase our understanding of socio-ecological behavior of the animals.

### Limitations of the study

Each of the two methods that we present here comes with a set of limitations, largely specific to our context and species, that we present here, together with suggestions for future improvements.

Our experimental method was designed for individual testing, which can be challenging to execute for animals living in larger groups. In our specific context this was addressed by enrolling additional people to help with a temporary separation of the individual being tested. It is important to note, however, that we acted upon the non-tested individuals, by luring them away with food, allowing the focal animal being tested the freedom to either engage with the task or leave. By doing so, we reduced (although we could not completely exclude) the levels of disturbance during testing. Yet, disturbance levels were noted down during coding, with dogs with high disturbance levels being excluded from the analyses. We recommend future studies to pay attention that tests are performed when there is the least disturbance possible in the environment and account for the possible exclusion of some animals later on. Nevertheless, the field environment, especially when testing social animals living in groups, meant a potential exposure of other pack individuals to our presence and to the test setting, which could have potentially biased their behavioral response when being tested in the future. Here, we initiated measures to counter this potential problem, by making sure individuals with prior exposure were tested after a time delay. While we think this solution was sufficient, we would still recommend that future studies keep detailed notes of the individuals that were previously exposed to the experimental set up and potentially include this information in the analysis. Group testing of personality traits has been used with free-ranging dog puppies by our group, showing good test-retest reliability at the individual level,^55^ thus this possibility could be explored further also with adult dogs, especially in situations where a potential group effect on the individuals’ reaction is considered acceptable.

A number of limitations are also linked to the observational data collection. During the observations, we recorded the positions of the animals when present within our study area. This presents certain limitations as only animals’ positions and behaviors that occur within our study area are recorded. While we assume that dogs keep the same social groups within and outside of our observed area, we cannot be certain of it. Therefore, it is likely that we missed some of the social interactions our subjects engaged in, which makes the assessment of familiarity toward other individuals impossible to determine accurately. Along the same line, some dogs may have larger exploration zones than those recorded within our study area. Nevertheless, such limitations are common to most field observations, and the overall high matching of behavioral responses when considering the experimental and observational methods suggest that such limitations did not have a strong impact on our main results. Yet, one possibility to keep into account in future studies would be to carry out focal observations of individual dogs/packs, thereby following their behavior also outside the study area. While this approach would produce a more accurate representation of how individuals use the area, it would be more time-consuming and it would reduce the number of individuals being observed, hence the final sample size. Given the trade-off between the amount of information obtained per subject and the number of subjects included in the behavioral observations, future studies should decide what they deem more important based on their specific research question.

Our approach of measuring conspecific-directed sociability did not account for distinction of familiarity between conspecifics, which led to unclear results. While in free living populations it is unlikely to know with certainty if animals living in the same area encountered each other before, we suggest that future studies should include measures of both proximities, as well as familiarity with each other. In this context unfamiliar dog presented during fake dog test might be more comparable to reaction toward individuals in the environment that are rarely encountered, as compared to other pack members. While our approach successfully measured human-directed sociability and exploration, as examples of behavior that commonly occur, the rarity of aggressive interactions did not allow us to investigate the aggressiveness trait. We recommend that for rare behaviors observational methods that permit focal observations, with continuous data collection should be applied.

## Resource availability

### Lead contact

Requests for additional details or resources should be directed to and will be fulfilled by the lead contact, Urša Blenkuš (ursa.blenkus@vetmeduni.ac.at).

### Materials availability

This study did not generate new unique materials.

### Data and code availability

• Data obtained from behavioral test battery and proximity scans has been deposited at FigShare (https://doi.org/10.6084/m9.figshare.29757116) and are publicly available as of the date of publication.
• This paper does not report original code.
• Any additional information is available from the lead contact upon request.

## Acknowledgments

The authors would like to thank Remco Folkertsma for his help and guidance with statistical analysis, Andreas Berghäenel for his useful suggestions on cross-context analysis and Ikhlass El Berbri for her logistics support and ongoing collaboration in Morocco. We would further like to thank the Morocco field group for all their hard work that made it possible to collect all the data (Svenja Capitain, Haytem Bouchri, Clemence Helleu, Jeremy Hardouin, Leo Hanon, Fiona Gaumard, Magdelena Juskaite, Giulia Cecchinato, Melissa Vanderheyden, Brenda Chaignon, Maiwen Braconnier, Camilla Mancassola, Manon Delaunay, Nina Truffaz, Claire Giraudet, and Valentina Napolitano). The study was funded by the 10.13039/501100002428Austrian Science Fund (10.13039/501100002428FWF) projects I-5052 to S.M.-P. and P-34749 to G.C.

## Author contributions

Conceptualization: U.B., S.M.-P., G.C., and F.R.; data curation: U.B., D.P., and C.A.; formal analysis: U.B.; funding acquisition: S.M.-P. and G.C.; investigation: U.B., D.P., C.A., and G.C.; methodology: U.B., G.C., and S.M.-P.; project administration: U.B., C.A., S.M.-P., and G.C.; resources: S.M.-P. and G.C.; supervision: S.M.-P., G.C., and F.R.; visualization: U.B.; writing – original draft: U.B.; writing – review and editing: S.M.-P., G.C., F.R., D.P., and C.A.

## Declaration of interests

The authors declare no competing interests.

## STAR★Methods

### Key resources table

**Table:** 

REAGENT or RESOURCE	SOURCE	IDENTIFIER
**Deposited data**		

Data	FigShare	https://doi.org/10.6084/m9.figshare.29757116

**Experimental models: Organisms/strains**		

Free-ranging dogs (*Canis familiaris*)	Souss-Massa region in Morocco	30°30’50 N, 9°41’00 W

**Software and algorithms**		

BORIS (Behavioral Observation Research Interactive Software)	BORIS software	https://www.boris.unito.it/
Map Marker	Map Marker App	https://www.mapmarker.app/
R Statistical Software	R Project	https://www.r-project.org/

### Experimental model and study participant details

#### Ethical statement

Ethical approval was obtained from the Ethical committee at the Agronomic and Veterinary Institute Hassan II (Comité d’Éthique de l’Institut Agronomique et Vétérinaire Hassan II - CESASPV) in Rabat, Morocco (Protocol number: CESASPV_2023_05), and further confirmed by the Ethical Commission of the University of Veterinary Medicine Vienna (Austria), following EU directives on animal testing. For the behavioural test battery, dogs were unrestrained, their participation in the test was voluntary, and they were able to leave at any time. During proximity scans, the observer did not interfere with the dogs’ behaviour in any way. All procedures were non-invasive and in accordance with EU Directive 2010/63/EU and approved by CESASPV to be in accordance with Morocco national legislation.

#### Study area and population

Research was conducted in the Souss-Massa region in Morocco (30°30’50 N, 9°41’00 W) with a free-ranging dog (*Canis familiaris*) population. They live in and around human settlements, in a highly touristic area around fishing villages, where they are exposed to daily interactions with humans. Dogs are free to roam and form groups but are largely dependent on humans for food provision. Since 2022 this population has been followed by a continuous monitoring program year-round. New dogs were first entered in our database as unknown individuals. When encountering them in the area at least three times over a period of two weeks, new dogs were given a unique name, noting additional information: sex, age, neutering status, body condition score, along with additional physical descriptions (i.e., tail length and shape, ear position, coat colour). Each dog was photographed from different angles to allow future recognition, and the general location, where the dog was usually encountered, was provided.

Proximity scans (PS), conducted between May 2022 and March 2024 were used in the current analyses. During this time, we individually identified 651 adult dogs within our study area. From the subset of identifiable dogs, a total of 201 dogs over 1 year of age were tested in the behavioural test battery (BTB) between November 2022 and March 2024. The complete BTB consisted of six subtests and three physiological sampling events conducted as part of the test. For the present study, we focused on the first three subtests (Human approach [HA], Fake dog [FD] and Novel object [NO]), that have an ecologically relevant meaning for a comparison with observations in dogs’ natural environment. The latter subtests (Pointing, Begging, and Tractability subtests) are meant to measure dogs’ socio-cognitive skills, hence less relevant for personality assessment. While all 201 dogs performed HA, 18 dogs left the experiment during the test or right after, therefore only 183 dogs were tested in FD and NO (below Table). As field conditions during testing are unpredictable, one of the coding variables considered was the presence of ‘disturbance’ during testing (defined as the time the dog’s focus shifted away from the test or the dog was no longer visible on camera). As this variable can impact the quality of the data, subtests, where disturbance presented more than 70% of the test duration, were excluded (below Table). For each dog tested, we considered the data coming from the PS in the 6 months leading up to the BTB. A 6-month window was chosen to ensure an equal time span with sufficient data across subjects, while limiting the possibility of personality changes over time.^56^^,^^57^ If an individual had no PS entries during this time window, it was excluded from further analysis (N=2, below Table). Since exploration of the environment (see below) during PS was calculated as distance between individuals’ locations from a central point (below Figure), to ensure a sufficient number of observations that would correctly represent individuals movement, we excluded the dogs that were observed in less than 10% of the total observation days (e.g. minimum 18 entries over a 180 day period). Dogs that had missing data for variables of interest were additionally excluded from further analysis (below Table).

**Table:** Number of dogs included in each subtest

Subtest	Tested	Disturbance <70%	Seen during PS		Final dataset (excluding missing data)	
			>0%	>10%		
HA	201	200	198	/	197	♀ 100 ♂97
FD	183	176	174	/	155	♀ 80 ♂75
NO	182	176	/	126	126	♀ 67 ♂59

HA, Human approach; FD, Fake dog; NO, Novel object; Tested, Number of dogs initially tested; Disturbance <70% - Number of dogs tested after removing dogs that had time disturbance coded for more than 70% of the subtest duration (dogs focus shifted away from the test or the dog was not visible on the camera); Seen during PS – Number of dogs included in the analysis after excluding dogs that were seen 0% (for HA and FD) or less than 10% (for NO) of the observation days; Final – final number of dogs included in the analysis after excluding dogs with missing data, separately presented for males and females.

**Figure:**
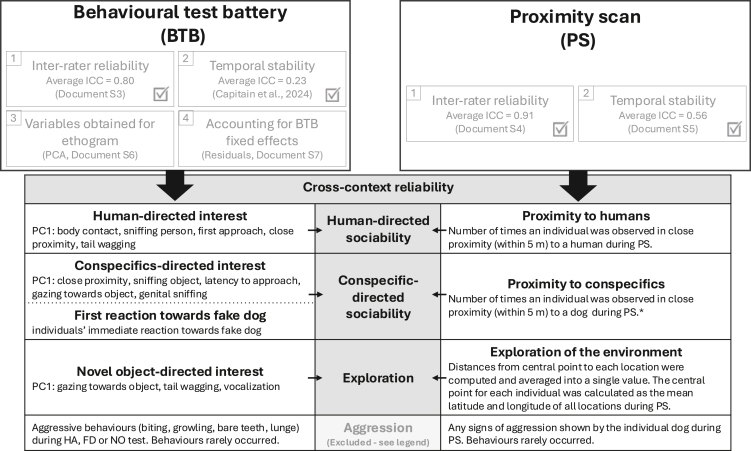
Visual presentation of the analysis Visual presentation of reliability measures (inter-rater reliability and temporal stability) and analysis (PCA and extraction of residuals) used to obtain final variables of interest from Behavioural test battery (BTB) and Proximity scans (PS) to perform cross-context reliability analysis for axes of interest (Human-directed sociability, Conspecific-directed sociability, Exploration, Aggression – due to low occurrence excluded from the analysis). PCA – Principal Component Analysis, PC1 – first dimension from PCA analysis. ∗We looked at three different measures of conspecific-directed sociability obtained from PS. Since all three measures presented us with same results, we found it redundant to present all three and are therefore presenting only the one that we consider most comparable to human-directed sociability.

### Method details

#### Behavioural test battery (BTB)

Detailed description of the whole test battery is presented in Capitain et al.^36^ Dogs were tested on the street, where they were usually observed – considered familiar home environment. Our intention was to test dogs individually, this was however not always possible, due to the unpredictable conditions such as humans interacting with the dogs during the test, too many dogs present in the area for only one distractor, female in heat followed by a group of males passing through the area … As we were beforehand aware of this issue,^36^ we tried to minimize disturbance by including three people in the experiment, adopting different roles: E1 – first experimenter (white Caucasian woman), interacting with the dog; E2 – second experimenter, assisting with test setup and preventing distractions from the environment; H – helper, minimizing the disturbance from the environment using food to lure other dogs out of sight from the testing area. The role of E1 was performed by 4 different female experimenters.

##### Human approach subtest (HA)

The test measured sociability towards humans, by observing at which stage of the subtest the dog approaches or leaves an unfamiliar human and the likelihood of it interacting with the human. The test was conducted over the following seven stages: a) E1 appeared approximately 5m away from the dog and stood quietly for 30 s, b) E1 started talking gently and making friendly gestures (gently clapping with hands, tapping with hands on the legs) towards the dog for 30 s, c) E1 slowly approached the dog to 2m taking approximately 30 s to do so, d) E1 stood at 2 m for 30 s, e) E1 approached the dog to approximately one body length and f) stood at this distance for 30 s, g) E1 crouched down for 30 s. E1 continuously talked to the dog from the stage b on (below Figure, Human approach). A dog that approached E1 in stage a was allocated the highest sociability score (score 7). For every additional stage that E1 had to initiate before a dog approached, the score was reduced by one (e.g. if a dog approached in stage a), it was given a score of 7, while a dog who approached in stage g received a score of 1). Dogs that never approached received the score of 0, while dogs who left during the experiment were given a negative score, with more negative scores reflecting earlier stages in which the dog left (e.g. score - 7 was given to dogs who left during stage a).

**Figure:**
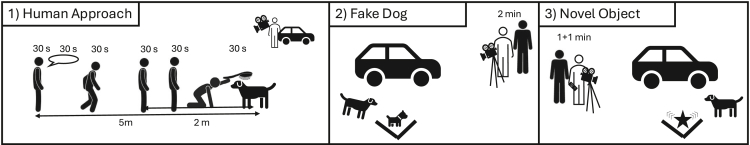
Experimental set up The set up of the first three subtests used in our behavioral test battery (BTB). Adapted from Capitain et al.^36^

##### Fake dog subtest (FD)

The test measured sociability towards a conspecific by observing the behaviours of the test subject towards a fake dog (“Giant Jack Russel Terrier”, brand: ‘Melissa & Douk’; wither height 28 cm). The use of fake dogs to evaluate a dogs’ reaction to unfamiliar conspecifics has been validated in other studies.^46^ Capitain et al.^36^ observed that during test-retest, dogs showed a reduced response towards the fake dog, when being exposed to it for the second time, suggesting that they perceive the fake dog as not being genuine faster the second time around. To ensure that we measured the dogs’ behaviour before they realised the dog was not real, we added the first reaction to the fake dog to the ethogram, a variable that captures the individuals’ initial reaction. A V-shaped barrier was used to hide the fake dog in such a way that E1 could guide the dog to approximately 1-2 m away from it before it became visible. E1 could use food to guide the dog to the starting position, however, as soon as the dog saw the fake dog (dogs head orientated towards FD), E1 stepped away. The dog’s response to the fake dog was observed for two minutes (above Figure, Fake dog).

##### Novel object subtest (NO)

The test measured the willingness of the animal to explore a novel object. The object was a 90–100 cm high foil balloon (four different balloons of similar size were used, replaced when inflation due to damage was no longer possible), fixed on a wooden stick and placed on top of a remotely controlled car (31x18x18 cm, Model: DEERC DE42RC). The same experimental set up was used as for the fake dog test. When the dog saw the novel object for the first time, E2 started moving the object back and forth, for 1 min, which was followed by another 1 min of the object staying still, bringing the whole duration of the test to 2 min (above Figure, Novel object).

While we tried to prevent the other dogs in the area (potential future subjects) from seeing the experimental set up, this was not always possible. Therefore, we noted all dogs that were present in the area while one subject was tested, and we only tested these dogs after at least one week if the dog observed the HA test beforehand and four weeks if the dog was present during the FD and/or NO test. Information related to prior exposure to the HA or FD/NO was available for 146 dogs. In total 31 out of these 146 dogs had observed a HA test before being tested themselves. We ran a linear model to assess the potential effect of prior exposure on the behaviour during the BTB, but found none (4.95 ± 15.23, t=0.33, df=1, p=0.75). One dog out of 146 was exposed to FD before being tested, and 7 dogs to NO. Due to the low number of dogs exposed to FD/NO, we do not consider this to be an issue in the current study. However, for future studies, we would consider keeping more detailed notes that could be included in the analysis if the number of dogs exposed to the test before would be too large.

#### Video coding of behavioural test battery

The tests were recorded using a digital camera (Sony HDR-CX405 Full HD Camcorder), either hand-held or placed on a tripod and operated by E2, and an action camera (Exprotrek 4K Ultra HD) fixed on E1’s chest. Videos were coded using BORIS^58^ (version 7.13.9), by two different coders. The ethogram developed by Capitain et al.^36^ was modified based on previously observed difficulties (removing behaviours with low coding reliability and reducing the number of modifiers). The activity level score and the first reaction towards the FD were added to the ethogram, adding to a final ethogram that contained 37 behaviours (Tables S1 and S2).

#### Proximity scans (PS)

Proximity scans are performed as part of our long-term population study. For the current study, data collected between May 2022 and March 2024 were considered. During this time, we conducted between 10 and 7 observations per week in our study area (total number of observations 720). Observations were performed randomly across the day (from sunrise to sunset). The observation area covered 41 ‘observation zones’ (total area size 763.38 ha, size of observation zone between 1.07 ha and 97.72 ha. The size of each observation zone varied because of their unique geographical and urban characteristics, which might have limited visibility (i.e. areas with fewer natural or artificial barriers, where visibility from a specific point of view was higher could be larger, whilst zones that had multiple barriers needed to be smaller to allow the observer to record reliably the location of each subject from a specific point). For each zone the observer noted down the time when scan was performed and the number of people present in the area (accurately up to ten, afterwards noted as ten or more people present). While moving between predefined observation zones, if observers noticed familiar dogs, they performed up to four extra observations per scan, increasing the scan to 45 ‘observation zones’. This provided us with additional information about our study population.

During a scan, the observer walked across the scan area, stopped once in each observation zone and conducted an observation by inserting each dog present in the observation area as a pin on a map, using Map Marker app (version 2.24.0_438) uploaded on a tablet (Samsung Galaxy SM-T395). Distances between the pins represent the distances between dogs. Reference points (e.g., houses, streets) visible on the map were used for accuracy. Map Marker provides the possibility to measure distance between two pins, which observers used, when in doubt if distance between two dogs was correctly presented. For each pin/dog the following information was added: the dog’s name, presence of people within 5 m, if the dog showed signs of aggression (growling, barking, or lunging towards conspecifics, humans, other animals, or cars), if a female in heat was present in the area or a mating occurred, whether the dog was feeding, or if the observer was unsure about the exact location of the dog. Since dogs often choose to follow people around, we noted if they had followed the observer into the area (observers presence influencing the presence of the dog). A detailed description of the behaviours recorded during the proximity scans is present in Table S3.

If the dog was not included in our data base (and therefore not individually recognizable), its age class (puppy, juvenile, adult) and sex (if observable) was noted, using predefined codes. A camera (Nikon Coolpix P900, 83x optical zoom wide, full HD) was used to take a picture of the dog, from the maximum distance possible (to not disturb the situation), using the maximum zoom. The picture was used for later identification (a time stamp and location allowing to reconstruct the scan situation).

Scans were conducted by 16 different observers over the course of the study period. Each observer went through a training phase (period of trainee shadowing experienced observers during the scan), until the trainee was able to recognize the majority of dogs on their own. At the end of the training phase 1-2 comparison scans were performed where the observer and the trainee performed a scan simultaneously without communicating with one another. Both scans were saved and afterwards used for inter-rater reliability calculation (see analysis and Document S4 for details).

#### Cross-context analysis between behavioural test battery and proximity scans

We assessed the cross-context validity of 4 personality traits: (I) conspecific-directed sociability, (II) exploration-avoidance (III) aggressiveness, (as defined by Reale et al.^2^), as well as (IV) human-directed sociability (a trait considered of interest for domestic species).

### Quantification and statistical analysis

To ensure the reliability of our two assessment methods, we first checked each for its inter-rater reliability (IRR). Interclass correlation coefficient (ICC) was used to calculate the reliability of continuous variables,^59^ with Kappa coefficient being used for categorical variables.^60^ A two-way random effects model with an absolute agreement estimate was used to assess the inter-rater reliability between the two coders. Kappa coefficients’ drastic correction for chance in situations where observed characteristic is very common or very rare,^60^ was problematic for PS variables (e.g. dogs were in majority of situations seen away from human, hence Kappa coefficients dramatically overcorrected inter-rater agreement for rare cases when dogs were seen in proximity to humans), therefore they were analysed using percentage agreement.^61^^,^^62^ For BTB, behaviours were assessed for each subtest. Behaviours that were included in all subtests were additionally summed up and assessed across the whole test. ICCs calculation was based on 10% of the videos (n=20) that were coded by two different observers. If behaviours did not reach our threshold (>0.70 ICC, moderate reliability^63^, then the behaviours were re-discussed and recoded on additional 5% of the videos (n=10). Two behaviours did not reach our threshold in the second round: ‘gazing towards E1’ was excluded, while ‘friendly was excluded for HA and NO subtest, but kept for FD as it reached the necessary threshold during this subtest (ICC = 0.88). ‘Mouthing’ and ‘whining’ occurred rarely, therefore small differences between observers resulted in low scores (0.00 and 0.07, respectively). Due to the low occurrence issue, we kept both behaviours in the analyses (detailed results in Tables S4 and S5). IRR for PS was assessed on comparison scans done by an experienced observer and a new trainee at the end of the training phase. Between April 2022 and August 2024, 16 observers and trainees performed 22 pair-comparisons. These scans were used to analyse agreement between the observers to (I) recognise the dogs, (II) assess the distances between the dogs, and (III) use the modifiers to provide additional information (unknown dog, known dog whose identity cannot be reliably confirmed, close to human, signs of aggression, feeding, following observer into the scan area, female in heat, mating occurrence, exact location of the dog present in the area unsure). As Kappa coefficient dramatically corrects for chance in situations where observed characteristic is very common (as discussed above), we used inter-rater agreement that was calculated as percentage of agreement between the two observers.^61^^,^^62^ All comparison scans reached moderate reliability (>0.70 ICC^63^; detailed results in Tables S6–S8).

PS was assessed for its temporal stability, by extracting the measures from the first and the last two months period (of the initial 6 months period of interest). Behaviours obtained between the two periods were compared, by using interclass correlation coefficient (ICC), commonly used method for estimating repeatability.^64^ Temporal stability for PS reached 0.49-0.64. In comparison with other studies (average temporal stability for personality measures of 0.43 in meta-analysis of 31 studies^57^), we considered our variables of good temporal stability. Detailed results presented in Table S9.

To reduce the number of variables from the BTB, a principal component analyses (PCA), was performed for each subtest (HA, FD, and NO) separately. For each analysis, behaviours that occurred rarely, were first joined with other similar behaviours. Variables that occurred for less than 10% of the dogs were removed from the analysis, as well as variables that failed normal distribution criteria. For all three subtests, the first dimension (PC1) appeared to be the most accurate representation of positive interest towards the stimuli (HA – body contact, sniffing person, first approach, close proximity, tail wagging; FD – close proximity, sniffing object, latency to approach, gazing towards object, genital sniffing; NO – gazing towards object, tail wagging, vocalization). These dimensions explained 33.23, 34.83, and 32.41 % of variability for the HA, FD, and NO subtests, respectively. Detailed analysis description and results are presented in Document S6. The first reaction towards the fake dog, as a categorical variable, was included separately in the analysis.

We ran three generalised linear models (GLM, one for HA, FD, and NO respectively, Table S14) including sex, temperature, body condition score, activity level during each test, and test specific characteristics (experimenter performing the test and activity level before starting the test for HA and, novel object used for NO) as predictors to extract residuals (PC1-residuals) used as a proxy for each personality score. These were used as predictors in the following analysis that addressed our main cross-context reliability question, for which we ran three models (one for human-directed sociability, conspecific-directed sociability and exploration, Table S18, Methods S1) that compared results from our two personality assessment methods. Our first binomial model looked at whether a positive interest towards humans during the HA test (HA-PC1-residuals) predicted proximity to humans during the PS, with proximity to humans included as a response variable composed by a two-column matrix: this included the number of observations in proximity to humans (1^st^ column) and the number of observations away from humans (2^nd^ column). To control for pseudoreplication, we included the individual as a random intercept effect. Looking at conspecific-directed sociability, we used the same binomial model structure, evaluating whether a positive interest towards the fake dog during the FD test (FD-PC1-residuals) predicted the proximity to other dogs during PS, with proximity to other dogs included as response variable composed by a two-column matrix (1^st^ column: number of observations in proximity to conspecifics; 2^nd^ column: number of observations away from conspecifics) and the first reaction towards the fake dog as fixed effect. To control for pseudoreplication, we included the individual as a random intercept effect. Finally, using a linear model we evaluated whether a positive interest towards the novel object during the NO test (NO-PC1-residuals) predicted exploration of the environment during PS, measured for each individual by computing the distance from a central point to each location (i.e. observation) and averaging it into a single value. The central point for each individual was calculated as the mean latitude and longitude of all locations during PS. Detailed analysis description of all models can be found in Document S7.

Statistical analyses and figures were performed in R (version 4.3.0; R Core Team, 2022). Inter-rater reliability of categorical variables was assessed using the kappa2 function from the irr package,^65^ providing a measure of agreement between raters. PCA was conducted using the FactoMineR package,^66^ with visualization and extraction of results facilitated by the factoextra package.^67^ Pre-analysis checks for PCA included the Kaiser-Meyer-Olkin Measure of Sampling Adequacy (KMO) and Bartlett's Test of Sphericity, performed using the KMO and cortest.bartlett functions from the psych^68^ package. Continuous variables were z transformed to a mean of zero and a standard deviation of one to ease the interpretation of the model coefficients.^69^ Statistical models were fitted using the glmer and lm functions from the lme4 package.^70^ Residual diagnostics included inspection of qq-plots and residuals plotted against fitted values to assess model assumptions. Collinearity among predictors was evaluated using generalized variance inflation factors (GVIFs) computed with the vif function from the car package.^71^ Model stability was assessed using DF-beta values,^72^ calculated with functions available in R.^73^ Overdispersion was tested using the overdisp.test() function, which indicated good to moderate stability of the models. Significance levels of the full-null model comparisons were determined using likelihood ratio tests.^74^ Confidence intervals for model estimates and fitted values were obtained using the emmeans package.^75^ The same package was used for post-hoc comparisons of the first reaction towards FD levels, using Tukey’s HSD method to correct p-values for multiple testing.
