# Supplementary material for: Personality traits in free-ranging dogs: Do experimental tests mirror natural behavior?
Source: iScience. 2025 Oct 25;28(11):113856. doi: 10.1016/j.isci.2025.113856 (PMC12661346; doi:10.1016/j.isci.2025.113856)
Supplement: Document S1. Tables S1–S18 [file mmc1.pdf]

iScience, Volume ■■

Supplemental information

Personality traits in free-ranging dogs: Do experimental tests mirror natural behavior?

Urša Blenkuš, Friederike Range, Debora Prince, Corisande Abiven, Giulia Cimarelli, and Sarah Marshall-Pescini

Document S1: Ethogram for Behavioural Test Battery

Table S1. Detailed Ethogram. The behaviours are presented by category (if applicable), with a detailed description and the unit in cursive in which they were measured. In addition, it is indicated in which subtest(s) the behaviour was coded and towards which modifier(s). Subtests include the Human approach (HA), Fake dog (FD), and Novel object (NO) subtest. Modifiers are the main experimenter (E1), the fake dog (FD), and the novel object (NO). If the behaviour was coded in general, this is marked with 'yes'. If the behaviour was not coded in the subtest, this is indicated with a minus (-).

Category	Behaviour	Definition	Applicable subtest		
			HA	FD	NO
Proximity	Close proximity *(Duration)*	Time within 1 body length of person: As soon as paw gets within 1 body length of person (actively or passively) TO last paw leaves distance of 1 body length of person	E1	FD	NO
Tail	Wagging *(Duration)*	Tail is actively moved from side to side (not just swinging with body movement)	E1	FD	NO
	Between the legs *(Duration)*	Tail is between the hind legs or below the belly	Yes	Yes	Yes
Gazing	Gazing *(Duration)*	Head oriented towards the stimulus	-	FD	NO
Vocalization	Barking *(Duration)*	Loud and short typical vocalization, which may be repetitive.	E1	FD	NO
	Growling *(Frequency)*	Actor snarls or growls at the specific receiver (only!) the actor's body posture being rigid. Mouth may be open, nose can be wrinkle.	E1	FD	NO
	Whining *(Duration)*	The dog making persistently whines.	E1	FD	NO
Displacement behaviours	Body Shaking *(Frequency)*	Dog's whole body starts moving rapidly, from side to side while the dog stands.	Yes	Yes	Yes
	Stretching *(Frequency)*	The whole body is stretched in various ways: forelegs may be bent down while the dog's back is arched; hind legs may be straightened while the head is held up high	Yes	Yes	Yes
	Yawning *(Frequency)*	The mouth is wide open, the neck is stretched.	Yes	Yes	Yes
	Nose /mouth licking	The mouth is slightly open, and the dog licks its upper lip or nose, on the right or on the left	Yes	Yes	Yes

	(Frequency)				
	Sniffing the ground *(Duration)*	Nose moving across the ground, head facing down instead of forward	Yes	-	-
Physical contact	Biting *(Frequency)*	The dog aggressively (!) bites another subject in the skin and fur. Includes bite-shaking. Aggressively meaning other signs of aggression such as growling, teeth baring, lunging have been shown during/before	E1	FD	NO
	Body contact *(Duration)*	Dog touches or is touched by person (or brush) with any parts of its body	E1	FD	NO
	Jumping *(Frequency)*	Dog jumps on or towards person: both front paws off the ground, both hind paws on the ground, front of body higher than back part of body, movement vertically towards person/wall/object	E1	FD	NO
	Licking *(Duration)*	Dog using its tongue to touch the person	E1	FD	NO
	Mouthing *(Duration)*	Dog takes part of the person/their clothes/object into its mouth without aggression	E1	FD	NO
	Sniffing person/object *(Duration)*	Nose close to the person/object	E1	FD	NO
	Pawing *(Frequency)*	Dog extends one paw towards person/object (distance less than 10 cm), the other is still on the ground	E1	FD	NO
Emotional reactions	Stand tall *(Duration)*	Straightens up to full height, with a rigid posture, ears erect and tail perpendicular or above the back. Standing or walking. May include raised hackles. May include T-pose towards fake dog (approaches another's shoulder/back/head and puts its head on it, typically forming a capital "T")	E1	FD	NO
	Bare teeth *(Frequency)*	Actor bares its canines, or generally curls the lips.	E1	FD	NO
	Lunge *(Frequency)*	Actor moves abruptly and rapidly towards recipient. Can but doesn't have to result in physical contact	E1	FD	NO
	Belly exposure *(Duration)*	To lie on the back showing the stomach holding the tail between the legs. The ears are held back and close to the head and the subject can raise a hind leg for inguinal presentation.	Yes	Yes	Yes
	Flee *(Frequency)*	To run away/ jump away (startle response) from stimuli with tail tucked between the legs and body ducked.	Yes	Yes	Yes

	Crouch *(Duration)*	Lowered posture bending the legs, arching the back, lowered tail	E1	FD	NO
	Risk assessment *(Duration)*	Back legs extended; nose reaches towards object	E1	FD	NO
	Play *(Duration)*	Jumping around with wagging tail while looking at person, play-bow (only with tail wagging, relaxed clumsy body posture)	E1	FD	NO
	Friendly *(Duration)*	Moves towards the receiver or stays in their vicinity in relaxed posture with ears pointed forward and the head pointed towards the receiver with the tail wagging at or above body line.	E1	FD	NO
Activity level	Activity level *(Binary)*	Inactive – if the dog is standing still or walking slowly for most of the test, while the dog needs a lot of encouragement to perform the tests.<br>Medium active – if the dog is walking for the most of the test, with very little or no running in between. The dog does stay still in one place, but when encouraged by the experimenter, moves without hesitation.<br>Very active – if the dog is walking or running around for most of the test. There are only some short periods when the dog is staying still.	Yes	Yes	Yes
Not visible	Not visible *(Duration)*	Out of camera view	Yes	Yes	Yes
Disturbance	Disturbance *(Duration)*	When behaviour is clearly directed at dogs/ other persons passing by/ distracting activities by E1/2 (e.g. feeding other dogs)/ other dog getting close enough to disturb the test	E1 / E2 / Person / Dogs		
Termination	Termination *(Occurrence)*	Termination of subtest or full test	Yes	Yes	Yes

Table S2. Ethogram for subtest-specific variables. The behaviours are presented for specific subtest to which they refer to (Human approach (HA), Fake dog (FD), and Novel object (NO) subtest), with a detailed description and the unit in cursive in which they were measured. In addition, it is indicated in which subtest(s) the behaviour was coded and towards which modifier(s). Modifiers are the main experimenter (E1), the fake dog (FD), and the novel object (NO). If the behaviour was not coded in the subtest, this is indicated with a minus (-).

Subtest-specific variables					
Subtest	Behaviour	Definition	Applicable subtest		
			HA	FD	NO
Human approach	First approach<br>*(Binary)*	Dog approaches E1 within 1 body length distance for the first time	E1	-	-
Fake dog	First reaction<br>*(Binary)*	First reaction towards the fake dog:<br>Mild avoidance - after seeing FD the dog steps to the side, gaze aversion, steps away, with other mild fear signals<br>Strong avoidance - after seeing FD the dog runs away while showing other fear signals, startle response<br>Fearful approach - low tail (can be low tail wagging) and/or risk assessment (back legs extended, nose reaches towards object)<br>Friendly approach - mid line or high relaxed tail wagging, relaxed posture<br>Rigid approach - high tail position, no tail wagging or stiff tail wagging, stiff body posture<br>Aggressive approach - signs of aggression: barking, biting, growling, showing teeth, snapping, lunging.<br>Freeze - after seeing FD the dog does not move from the initial position and does not move its body.<br>Uncertain - not possible to classify, because of external circumstances that made it impossible to compare it with the other dogs or the dog does not show any reaction towards FD.	-	FD	-
	Latency to approach fake dog<br>*(Duration)*	Time until the dog actively gets into less than 2 body lengths from the fake dog from first seeing it	-	FD	-

	Genital sniffing<br>*(Frequency)*	To sniff the genital area of the fake dog	-	FD	-
Novel object	Latency to approach novel object<br>*(Duration)*	Time until the dog actively gets into less than 2 body lengths from the object from first seeing it	-	-	NO

Document S2: Ethogram for proximity scans

Table S3: Detailed Ethogram of behaviours recorded during proximity scans

Behaviour	Description
Human	A dog is within 5 meters from a human (including the observer)
Pet	A dog who is obviously owned (e.g. with a collar or kept on lead)
Feeding	The dog is eating something
Heat	For the female who is in oestrus (vaginal bleeding, vulvar swelling, vulvar presentation, tail deviation).
Mating	For the 2 individuals involved in the mating (2 individuals in the copulatory tie). Always add heat before mating.
Following observer	When a dog has followed you to a new scan area from the previous one. You only add this label at the second scan area (and following ones) but not at the very first one where you saw the dog.
Uncertain location	If you see a dog present in the scan area but after having done the scan (that is, you are not sure where the dog was in the moment of the scan).
Aggressive interaction	For all individuals within 2 meters from an individual showing aggression.
Unsure	You see a dog and you know that you know the dog but don't remember the name or you cannot be sure who it is (e.g. if there are 2 similar dogs with similar characteristics).
Unknown	You see a dog that you are sure it is not in our data base. In case you manage to make a picture, the dog can be later on added to the data base and the name added to the marker.
Adult-male<br>Adult-female<br>Adult-NA	Try to identify sex of the unknown adult. If not possible identify only that it is unknown adult.
Juvenile-male<br>Juvenile-female<br>Juvenile-NA	Try to identify sex of the unknown juvenile. If not possible identify only that it is unknown juvenile.<br>If it is a known litter then add the mothers name and if you are able to identify the juvenile add its identification number.
Pup-male<br>Pup-female<br>Pup-NA	Try to identify sex of the unknown puppy. If not possible identify only that it is unknown puppy.<br>If it is a known litter then add the mothers name and if you are able to identify the puppy add its identification number.<br>If there is more than 1 puppy all together (e.g. when still very young), use only 1 marker and add the number of puppies in the description.
Den	A location where young puppies are. If you know who is the mother, add her name. Add the number of puppies in the den in the description.
None	Marker label for when a scan area is empty.

Document S3: Inter-Rater Reliability for Behavioural Test Battey

Table S4. Detailed inter-rater reliability outcome for BTB (n=20) Presented as interclass coefficient (ICC) result for each individual subtest of interest in this study and summed up across all the six subtest of BTB. Modifier: HA – behaviour towards human during Human Approach subtest; FD – behaviour towards Fake dog during Fake dog subtest; NO – behaviour towards Novel object during Novel object test; Any – behaviour occurred during any of the subtests, recipient of the behaviour not specified. Behaviours that reached our threshold (high ICC score >0.70) are presented in bold.

Variable	Modifier	ICC	Lower CI	Upper CI	F	Df1	Df2	p
Close proximity	HA	**0.92**	0.81	0.97	2.36	19.00	19.26	0.00
	FD	**0.94**	0.86	0.98	3.36	19.00	19.83	0.00
	NO	**0.96**	0.90	0.98	4.82	19.00	19.57	0.02
Tail wagging	HA	**0.93**	0.84	0.97	2.83	19.00	20.00	0.00
	FD	**0.76**	0.49	0.90	7.55	19.00	19.77	0.00
	NO	**0.99**	0.98	1.00	2.81	19.00	18.24	0.00
Tail between the legs	Any	**0.88**	0.73	0.95	16.08	19.00	19.99	0.00
Gazing[3]	HA	0.64	0.12	0.86	6.99	19.00	6.49	0.01
	FD	**0.90**	0.75	0.96	2.01	19.00	17.24	0.00
	NO	**0.88**	0.73	0.95	1.54	19.00	19.52	0.00
Barking	Any	**1.00**	1.00	1.00	1.99	19.00	19.42	0.00
Growling[1]	Any	**0.90**	0.65	0.97	17.54	9.00	9.18	0.00
Whining[1]	Any	0.07[2]	-0.55	0.64	1.15	9.00	9.23	0.00
Body Shaking	Any	**0.92**	0.81	0.97	2.42	19.00	20.00	0.00
Stretching	Any	**0.85**	0.13	0.90	5.07	9.00	9.80	9.80
Yawning	Any	**0.86**	0.69	0.94	13.20	19.00	19.51	0.00
Nose /mouth licking	Any	**0.86**	0.66	0.95	16.22	19.00	13.95	0.00
Sniffing the ground	Any	**0.87**	0.70	0.95	1.45	19.00	19.90	0.00
Biting	Any	NaN	NaN	NaN	NaN	19.00	NaN	NaN
Body contact	Any	**0.98**	0.87	0.99	1.84	19.00	43.91	0.00
Jumping	Any	**0.92**	0.81	0.97	2.37	19.00	20.00	0.00
Licking	Any	**0.82**	0.61	0.92	1.05	19.00	19.66	0.00
Mouthing	Any	0.00[2]	-0.42	0.43	1.00	19.00	19.00	0.50
Sniffing person/object	Any	**0.70**	-0.02	0.89	10.79	19.00	3.29	0.03
Pawing	Any	**0.85**	0.65	0.94	1.18	19.00	19.84	0.00
Stand tall[1]	Any	NaN	NaN	NaN	NaN	9.00	NaN	NaN
Bare teeth	Any	NaN	NaN	NaN	NaN	19.00	NaN	NaN
Lunge	Any	NaN	NaN	NaN	NaN	19.00	NaN	NaN
Belly exposure	Any	**0.98**	0.94	0.99	8.07	19.00	20.00	0.73
Flee	Any	**0.79**	0.54	0.91	8.50	19.00	19.97	0.00
Crouch	Any	**0.74**	0.43	0.89	7.89	19.00	14.71	0.00
Risk assessment	Any	**0.75**	0.47	0.89	6.74	19.00	19.48	0.00
Play	Any	**0.97**	0.93	0.99	7.21	19.00	20.00	0.00
Friendly[1]	Any	0.23[3]	-0.44	0.73	1.58	9.00	9.45	0.25
	FD	**0.88**	0.60	0.97	17.25	9.00	9.20	0.00
Activity level[1]	Any	**0.84**	0.43	0.96	14.86	9.00	6.82	0.00
Not visible or Disturbance[1]	Any	**0.71**	0.01	0.91	8.52	9.00	4.29	0.02
Termination	Any	**0.90**	0.77	0.96	18.68	19.00	20.00	0.00
First approach	HA	**0.99**	0.98	1.00	288.90	19.00	19.00	0.00
Latency to approach fake dog	FD	**0.72**	0.42	0.88	6.06	19.00	19.30	0.00
Genital sniffing	FD	**0.80**	0.56	0.91	9.00	19.00	19.96	0.00
Latency to approach novel object	NO	**0.77**	0.52	0.90	8.15	19.00	19.66	0.00

[1] *Results presented from second recoding on a set of n=10 dogs, due to low ICC score during first test.*
[2] *Low result, due to one very short occurrence, after final inspection still included in the analysis.*
[3] *Excluded from the further analysis.*

Table S5. Detailed inter-rater reliability outcome for BTB (n=10) Presented as Kappa coefficient (unweighted to treat all the disagreements equally, Grant et al., 2017) for FD First reaction, as categorical data. Kappa coefficient bellow 0.20 is considered poor, between 0.21 and 0.40 fair, between 0.41 and 0.60 moderate, between 0.61 and 0.80 good and above 0.81 very good (Flight & Julious, 2015). Modifier: FD – behaviour towards Fake dog during Fake dog subtest.

	Modifier	Kappa	p	z
First reaction	FD	**0.76**	0.00	5.35

Document S4: Inter-Rater Reliability for Proximity Scans

Table S6: Inter-rater agreement for PS assessing dog recognition score, as percentage agreement between the comparison pairs (entries in each observation area were compared, and if both observers recoding the same dog this was considered agreement, if only one observer recorded the dog's presence this was considered disagreement). Comparison pairs that reached our threshold (good agreement >75%) are presented in bold.

Date	Observer 1	Observer 2	**Percentage Identified**
23.4.2022	MJ	GC	**87.69**
4.5.2022	HB	UB	**86.92**
7.6.2022	MJ	SC	**79.1**
8.6.2022	SC	GC	**80.85**
12.7.2022	MJ	MD	**88.89**
13.7.2022	MD	SC	**82.43**
21.1.2023	LH	UB	**81.42**
22.1.2023	JH	UB	**98.08**
23.1.2023	LH	JH	**98.2**
30.1.2023	CH	JH	**96.67**
31.1.2023	CH	LH	**92.68**
22.2.2023	CH	FG	**95.97**
23.2.2023	FG	LH	**90.97**
19.11.2023	DP	UB	**88.06**
5.12.2023	UB	NT	**95.97**
10.12.2023	NT	CA	**85.91**
31.3.2024	VN	CA	**81.48**
1.4.2024	LH	VN	**92.86**
16.4.2024	LH	CM	**78.74**
18.4.2024	CM	UB	**87.76**
28.8.2024	LH	LS	**85.81**
31.8.2024	CA	LS	**83.33**

Table S7: Inter-rater agreement for PS data assessing modifiers performed on observations where dogs identity was correctly identified by both observers. Due to the very low occurrence of modifiers, our data did not have sufficient variability to calculate agreement between each comparison pair separately, therefore comparison was done across all the scan comparison entries (2738 entries), as percentage agreement - number of agreements divided by number of agreements and disagreements (unk – unknown dog; uns – known dog whose identity cannot be reliably confirmed; hum – close to human; agg – signs of aggression; fee – feeding; foo – following observer into the scan area; hea – female in heat; mat – mating occurrence; unl – exact location of the dog present in the area unsure). All the modifiers reached our threshold (good agreement >75%), presented in bold.

Modifier	Percentage
unk	**97.44**
uns	**99.60**
hum	**88.49**
agg	**99.71**
fee	**99.71**

foo	**98.17**
hea	**99.53**
mat	**100.00**
unl	**96.09**

Table S8: Inter-rater agreement for distances between dogs, as interclass correlation coefficient between the comparison pairs for dogs that were reliably observed by both observers. ICC below 0.5 as poor, between 0.50 and 0.75 as moderate, between 0.75 and 0.90 as good and above 0.90 as excellent (Koo & Li, 2016). Comparison pairs that reached our threshold (good agreement >75%) are presented in bold.

date	Observer 1	Observer 2	ICC	Lower CI	Upper CI	F	Df1	Df2	p
23.04.2022	GC	MJ	**0.79**	0.70	0.85	8.76	102.00	94.06	0.000
04.05.2022	UB	HB	**0.94**	0.92	0.96	33.15	178.00	178.81	0.000
07.06.2022	SC	MJ	**0.94**	0.92	0.95	30.94	194.00	193.48	0.000
08.06.2022	GC	SC	**0.92**	0.90	0.94	24.48	311.00	311.10	0.000
12.07.2022	MD	MJ	**0.96**	0.94	0.97	47.28	103.00	103.52	0.000
13.07.2022	SC	MD	**0.93**	0.91	0.95	29.59	136.00	136.71	0.000
21.01.2023	UB	LH	**0.97**	0.96	0.98	65.23	184.00	128.56	0.000
22.01.2023	UB	JH	**0.98**	0.98	0.99	117.05	227.00	228.00	0.000
23.01.2023	JH	LH	**0.98**	0.98	0.99	118.13	234.00	189.45	0.000
30.01.2023	JH	CH	**0.88**	0.83	0.91	15.24	139.00	137.27	0.000
31.01.2023	LH	CH	**0.87**	0.79	0.91	15.30	106.00	52.23	0.000
22.02.2023	FG	CH	**0.96**	0.94	0.97	44.97	248.00	177.54	0.000
23.02.2023	LH	FG	**0.94**	0.92	0.95	33.63	251.00	191.52	0.000
19.11.2023	UB	DP	**0.95**	0.94	0.96	41.94	278.00	277.18	0.000
05.12.2023	NT	UB	**0.92**	0.87	0.95	30.09	371.00	33.62	0.000
10.12.2023	CA	NT	**0.73**	0.66	0.78	6.76	330.00	170.09	0.000
31.03.2024	CA	VN	**0.87**	0.83	0.90	14.17	168.00	168.84	0.000
01.04.2024	VN	LH	**0.92**	0.88	0.94	23.06	117.00	114.63	0.000
16.04.2024	CM	LH	**0.97**	0.96	0.98	72.04	200.00	192.42	0.000
18.04.2024	UB	CM	**0.88**	0.84	0.90	15.54	290.00	209.60	0.000
28.08.2024	LS	LH	**0.98**	0.98	0.98	101.91	342.00	342.64	0.000
31.08.2024	LS	CA	**0.93**	0.90	0.95	27.22	199.00	139.79	0.000

Document S5: Temporal Stability of Proximity Scans

Table S9. Detailed temporal stability outcome for PS (n=113) presented as interclass correlation coefficient (ICC), obtained by comparing two-month period of 6 to 4 months before and 2 to 0 months before BTB. Behaviours that reached our threshold (ICC score >0.50) are presented in bold.

Variable	Modifier	ICC	Lower CI	Upper CI	F	Df1	Df2	p
Humans within 5m	Number of times an individual was observed in close proximity (within 5 m) to a human during PS.	**0.64**	0.40	0.78	5.79	112	20.95	<0.001
Sum known dogs within 5m	Number of times an individual was observed in close proximity (within 5 m) to a dog during PS.	**0.54**	0.38	0.66	3.57	112	79.66	<0.001
Distance between individual GPS locations	The central point for each individual was calculated as the mean latitude and longitude of all individuals locations during PS. Distances from central point to each location were then computed and averaged into a single value.	**0.49**	0.34	0.62	2.91	112	112.12	<0.001

Document S6: Principal Component Analysis for Behavioural Test Battery

Table S10: Behaviours excluded from the analysis due to low occurrence – observed in less then 10% of the dogs (<24, total number of dogs tested in our study are and surrounding is 237). Table is presenting the number of dogs that showed excluded behaviours during each of the subtests (unit is initiated in the brackets F – Frequency, D – Duration, F+D – Frequency and duration). Behaviour not excluded for specific subtest is indicated with grey.

**Behaviour**	**Human approach**	**Fake dog**	**Novel object**
Biting	0 *(F)*	1 *(F)*	0 *(F)*
Stand tall	0 *(F+D)*	19 *(D)*	5 *(F+D)*
Bare Teeth	0 *(F)*	0 *(F)*	0 *(F)*
Lunge	1 *(F)*		0 *(F)*
Belly exposure	5 *(F+D)*	0 *(F+D)*	0 *(F+D)*
Tail between the legs	18 *(D)*	19 *(D)*	24
Risk assessment	2 *(F+D)*		15 *(F+D)*
Crouch	10 *(D)*	13 *(D)*	14 *(D)*
Play	4 *(F+D)*		0 *(F+D)*
Barking	6 *(D)*	10 *(D)*	24 *(D)*
Whining	8 *(D)*	1 *(D)*	1 *(D)*
Vocalization<br>- Barking<br>- Growling<br>- Whining	16 *(F)*	12 *(F)*	
Jumping		0 *(F)*	0 *(F)*
Pawing		0 *(F)*	0 *(F)*
Licking		1 *(F+D)*	0 *(F+D)*
Mouthing		2 *(F+D)*	0 *(F+D)*
Shaking			0 *(F)*
Stretching			3 *(F)*
Body contact			2 *(F+D)*
Sniffing object			16 *(F+D)*

Table S11. Results from PCA for Human approach test (KMO=0.78). If behaviour was observed rarely, it was combined with similar behaviours: fear signals (sum of tail between the legs, flee, and crouch), contact seeking (sum of jumping and pawing). Behaviours that failed normal distribution criteria (failed skewness criteria even after log transformation) were excluded.

	Interaction (Dimension 1)	Sniffing ground (Dimension 2)	Stress (Dimension 3)
Close proximity towards E1	0.69	0.06	-0.03
Tail wagging towards E1 *(Duration)*	0.74	0.10	0.10
Tail wagging towards E1 *(Frequency)*	0.57	0.02	0.24
Nose /mouth licking *(Frequency)*	0.52	0.06	0.24
Body contact *(Duration)*	0.77	0.07	-0.15
Body contact *(Frequency)*	0.82	0.16	-0.10
Sniffing person/object *(Duration)*	0.76	0.11	-0.23
Sniffing person/object (Frequency*)*	0.84	0.10	-0.14
First approach	0.70	0.09	-0.10
Body Shaking *(Frequency)* *(1)*	0.43	-0.08	0.44
Stretching *(Frequency)* *(1)*	0.24	-0.26	0.75
Yawning *(Frequency)* *(1)*	0.02	-0.29	0.70
Sniffing the ground *(Duration)* *(1)*	-0.28	0.91	0.26
Sniffing the ground *(Frequency)* *(1)*	-0.29	0.91	0.27
Fear signals *(Frequency)* *(1)*<br>- Tail between the legs *(Frequency)*<br>- Flee *(Frequency)*<br>- Crouch *(Duration)*	-0.07	0.03	-0.02

Contact seeking *(Frequency)* (1)<br>- Jumping *(Frequency)*<br>- Pawing *(Frequency)*	0.50	0.10	0.03
Variance percent	33.23	11.83	10.16
Eigen value	5.31	1.89	1.62

(1) Variable log transformed to improve normal distribution criteria

Table S12. Results from PCA for Fake dog test (KMO=0.76) If behaviour was observed rarely, it was combined with similar behaviours: self-directed behaviours (sum of body shaking, stretching and yawning), fear signals (sum of tail between the legs, flee, and crouch), aggressive behaviours (sum of stand tall and lunge), affiliative behaviours (sum of play and friendly). Behaviours that failed normal distribution criteria (failed skewness criteria even after log transformation) were excluded.

	Close investigation (Dimension 1)	Fearful assessment (Dimension 2)	Tail wagging (Dimension 3)
Close proximity towards FD *(Duration)*	0.87	-0.02	-0.21
Sniffing person/object *(Duration)*	0.84	-0.23	-0.28
Sniffing person/object (Frequency)	0.86	-0.18	-0.20
Gazing towards FD *(Duration)*	0.73	0.28	0.22
Gazing towards FD *(Frequency)*	0.35	0.56	0.39
Latency to approach fake dog *(Duration)*	-0.64	0.18	0.29
Genital sniffing *(Frequency)*	0.74	-0.29	-0.23
Tail wagging towards FD *(Duration)* (1)	0.56	-0.30	0.65
Tail wagging towards FD *(Frequency)* (1)	0.58	-0.22	0.65
Nose /mouth licking *(Frequency)* (1)	0.12	-0.03	0.34
Body contact *(Duration)* (2)			
Body contact *(Frequency)* (2)			
Risk assessment *(Duration)* (1)	0.42	0.76	-0.15
Risk assessment *(Frequency)* (1)	0.43	0.75	-0.11
Self-directed behaviours *(Frequency)* (1)<br>- Body Shaking *(Frequency)*<br>- Stretching *(Frequency)*<br>- Yawning *(Frequency)*	-0.04	-0.06	0.25
Fear signals *(Frequency)* (1)<br>- Tail between the legs *(Frequency)*<br>- Flee *(Frequency)*<br>- Crouch *(Duration)*	-0.11	0.49	0.11
Aggressive behaviours *(Frequency)* (2)<br>- Stand tall *(Frequency)*<br>- Lunge *(Frequency)*			
Affiliative behaviours *(Duration)* (2)<br>- Play *(Duration)*<br>- Friendly *(Duration)*			
Affiliative behaviours *(Frequency)* (2)<br>- Play *(Frequency)*<br>- Friendly *(Frequency)*			
Variance percent	34.83	15.10	11.30
Eigen value	4.88	2.11	1.58

(1) Variable log transformed to improve normal distribution criteria
(2) Due to not reaching normal distribution criteria, variable removed from the analysis.

Table S13. Results from PCA for Novel object test (KMO=0.57) If behaviour was observed rarely, it was combined with similar behaviours: fear signals (sum of tail between the legs, flee, and crouch), vocalization (sum of barking, growling and whining). Behaviours that failed normal distribution criteria (failed skewness criteria even after log transformation) were excluded.

	Proactive investigation (Dimension 1)	Fearful assessment (Dimension 2)	Stressful approach (Dimension 3)	Stress (Dimension 4)
Nose /mouth licking *(Frequency)*	-0.13	0.37	0.21	0.78
Gazing towards NO *(Duration)*	0.64	0.40	0.42	-0.06
Gazing towards NO *(Frequency)*	0.35	0.56	0.59	0.09
Latency to approach the novel object *(Duration)*	-0.26	0.72	-0.48	0.06
Fear signals *(Frequency)*<br>- Tail between the legs *(Frequency)*<br>- Flee *(Frequency)*<br>- Crouch *(Duration)*	0.24	0.49	0.14	-0.59
Close proximity towards NO *(Duration)* (1)	0.42	-0.62	0.54	0.05
Tail wagging towards NO *(Duration)* (1)	0.88	-0.10	-0.38	0.16
Tail wagging towards NO *(Frequency)* (1)	0.85	-0.06	-0.36	0.19
Yawning *(Frequency)* (2)				
Vocalization *(Frequency)* (1)<br>- Barking *(Frequency)*<br>- Growling *(Frequency)*<br>- Whining *(Frequency)*	0.76	0.10	-0.26	-0.09
Variance percent	32.41	19.94	16.08	11.64
Eigen value	2.92	1.79	1.44	1.04

(1) Variable log transformed to improve normal distribution criteria
(2) Due to not reaching normal distribution criteria, variable removed from the analysis.

Document S7: Description of models used for comparison between Behavioural Test Battey and Proximity Scans

Methods S1: Detailed description of the cross-context validity analyses

To control for influence of fixed effects that could impact results obtained from PC1 related to BTB, we ran three generalised linear models (Table S14). We extracted the residuals from the model and used it as a proxy for each personality score measured from BTB that was included in the second model, where we compared personality score from BTB with personality score obtained from PS.

Results of the three control models (Table S14) for HA, FD, and NO are presented in Table S15, Table S16, and Table S17, respectively.

A full-null model comparison for HA test of predictors with possible effects (ANOVA, test = "F") strongly supported the inclusion of test predictors in the model ($F = 3.545$ $df = 8$, $p = < 0.001$, results for each predictor presented in Table S15).

A full-null model comparison for FD test of predictors with possible effects (ANOVA, test = "F") did not show statistically significant predictors of the model ($F = 2.395$, $df = 2$, $p = 0.094$, results for each predictor presented in Table S16).

A full-null model comparison for NO test of predictors with possible effects (ANOVA, test = "F") did not show statistically significant predictors of the model ($F = 1.932$, $df = 5$, $p = 0.094$, results for each predictor presented in Table S17).

To analyse cross-context reliability between BTB and PS we used three main models (Table S18). To address our first question of human-directed sociability, we used Generalized Linear Mixed-Effects Models (GLMER) with binomial distribution, to analyse whether positive interest towards humans during HA test, that was obtained from HA PC1 analysis (first dimension was considered as most accurate assessment of positive interest towards humans) predicts proximity to humans during PS, that was measured as number of times the dog has been seen close to human or not, each observation considered as a trial. Detailed presentation of the model in Table S18, Model 1.2.

Second question of conspecific-directed sociability was addressed using the same model, with response variable being considered the number of times the dog has been seen close to other dogs during PS or not, each observation considered as a trial. For fixed effects, we considered two factors; positive interest towards fake dog during FD test, that was obtained from FD PC1 analysis (first dimension was considered as most accurate assessment of positive interest towards fake dog) and first reaction of the dog towards fake dog (mild avoidance (n=65), strong avoidance (n=3), fearful approach (n=20), friendly approach (n=28), rigid approach (n=31), aggressive approach (n=2), freeze (n=6), uncertain(n=19)). Due to low occurrences of some first reaction categories, we joined strong and mild avoidance into avoidance, aggressive approach was summed together with rigid approach, and freezing was summed with fearful approach. Uncertain was removed from the analysis, resulting in final sample of 155 dogs. Detailed presentation of the model in Table S18, Model 2.2.

Last model looked at exploration, which we analysed using linear model (LM). We predicted that positive interest towards novel object during NO test, which was obtained from NO PC1 analysis (first dimension was considered as most accurate assessment of positive interest towards novel object), will predict exploration of the environment, measured as average distance between observation points during PS. To account for the differences in the number of times the dog has been seen, we included this variable as vector of weights in the model. Detailed presentation of the model in Table S18, Model 3.2.

Results of the three main models (Table S18) for human-directed sociability, conspecific-directed sociability, and exploration are presented in main text, under results.

Table S14: Details on the models from PC1 analysis used to extract residuals from BTB test

	Model 1.1 - HA	Model 2.1 - FD	Model 3.1 - NO
Control model[3]	Generalized linear model (GLM)	Generalized linear model (GLM)	Generalized linear model (GLM)
Sample size	197	155	126
Response	PC1 for HA	PC1 for FD	PC1 for NO
Key fixed effects for control model	- body condition class,<br>- temperature during BTB[1],<br>- activity level during HA[1],<br>- sex,<br>- experimenter performing BTB<br>- activity level before starting the test	- body condition class[2],<br>- temperature during BTB[1],<br>- activity level during FD[2],<br>- sex	- body condition class[2],<br>- temperature during BTB[1],<br>- sex,<br>- novel object used during experiment
Significance check for control model	Full-Null model comparison (ANOVA Chi-square)	Full-Null model comparison (ANOVA Chi-square)	Full-Null model comparison (ANOVA Chi-square)

[1] z transformed variables
[2] considered unbalanced and removed from the model
[3] model used to create residuals on the respective PC1 scores, to create personality scores that are controlled for influence of effects from BTB

Table S15: Results of the model of residuals for fixed effects for human-directed sociability on BTB (estimates and standard errors, together with confidence limits, and significance tests)

	Estimate	St err	Lower CI	Upper CI	t	df	p[1]
Intercept	120.58	36.82	48.42	192.74			[2]
Body condition class	0.22	10.99	-21.31	21.76	0.00	1	0.983
Temperature [3]	2.53	6.02	-9.26	14.32	0.19	1	0.667
HA activity level[4]	20.38	6.39	7.86	32.90	10.39	1	**0.001**
State class	-32.03	12.62	-56.76	-7.31	6.64	1	**0.010**
Sex M[5]	-9.25	12.62	-33.98	15.48	0.56	1	0.453
E1 DP[6]	5.05	16.82	-27.92	38.03	14.97	3	**0.002**
E1 FG[6]	48.99	20.92	7.99	89.99			
E1 UB[6]	48.45	15.28	18.51	78.38			

[1] the indicated significance test referees to the overall effect of the predictors
[2] not indicated because of being of very limited interpretation
[3] z transformed to a mean of 0 and standard deviation of 1; mean and sd of the original variable were 18.29 and 4.11 °C, respectively
[4] z transformed to a mean of 0 and standard deviation of 1; mean and sd of the original variable were 1.98 and 0.67, respectively
[5] referenced based on female
[6] reference based on Experimenter CA

Table S16: Results of the model of residuals for fixed effects for conspecific-directed sociability on BTB (estimates and standard errors, together with confidence limits, and significance tests)

	Estimate	St err	Lower CI	Upper CI	t	df	p
Intercept	32.47	7.14	18.47	46.46			[1]
Temperature[2]	-10.09	5.15	-20.19	0.00	3.87	1	0.052
Sex M[3]	-11.01	10.27	-31.14	9.13	1.17	1	0.286

[1] not indicated because of being of very limited interpretation
[2] z transformed to a mean of 0 and standard deviation of 1; mean and sd of the original variable were 18.529 and 4.047, respectively
[3] referenced based on female

Table S17: Results of the model of residuals for fixed effects for on BTB for Exploration (estimates and standard errors, together with confidence limits, and significance tests), BE – bee, BD – birthday, BF – butterfly, CE - celebration

	Estimate	St err	Lower CI	Upper CI	t	df	p[1]
Intercept	-6.49	5.18	-16.65	3.67			[2]
Temperature[3]	-2.74	2.47	-7.57	2.09	1.29	1	0.256
Sex M[4]	-4.38	3.49	-11.22	2.46	1.64	1	0.200
Novel object BD[5]	-6.16	9.30	-24.39	12.06	3.24	3	0.356
Novel object BF[5]	3.59	5.33	-6.86	14.04			
Novel object CE[5]	4.20	7.47	-10.44	18.84			

[1] the indicated significance test referees to the overall effect of the predictors
[2] not indicated because of being of very limited interpretation
[3] z transformed to a mean of 0 and standard deviation of 1; mean and sd of the original variable were 19.278 and 4.212, respectively
[4] referenced based on female
[5] reference based on BE

Table S18: Details on the models addressing cross-context reliability (comparison between BTB and PS)

	Model 1.2 – Human-directed sociability	Model 2.2 – Conspecific-directed sociability	Model 3.2 - Exploration
Model	Generalized Linear Mixed-Effects Models (GLMM; binomial residual error structure with logit-link transformed response)	Generalized Linear Mixed-Effects Models (GLMM; binomial residual error structure with logit-link transformed response)	Linear model (LM; with number of times the dog was seen in 6 months PS period considered as vector of weights)
Sample size	197	155	126
Response	Response matrix: seen close to human or not during PS on a trial basis	Response matrix: seen close to other dogs or not during PS on a trial basis	Average distance between observation points[2]
Key fixed effects	- Residuals obtained from PC1 for HA[1]	- Residuals obtained from PC1 for FD[1]<br>- First reaction towards fake dog	- Residuals obtained from PC1 for NO[1]
Further fixed effects	/	/	- Activity level during NO
Control optimizer	bobyga	bobyga	/
Significance check	/	Full-Null model comparison (ANOVA Chi-square)	Full-Null model comparison (ANOVA Chi-square)

[1] z transformed variables
[2] log transformed variables
